# Morphine Habit Cured*Postscript to a personal letter to the editor.

**Published:** 1895-04

**Authors:** Wm. Keaney

**Affiliations:** De Soto, Mo.


### MORPHINE HABIT CURED.*

Wm. Keaney, M. D., De Soto, Mo.

I have been talking within the last two hours to a man æt. forty-eight, who has for nearly fifteen years been taking at least ten grains of Morphine per diem. My father has cured him of the habit with ONE dose of Sulph.$^{cm.}$ (Swan). He has been cured now for eight months; gone up in weight from 135 to 195 pounds. The unpleasant symptom that remains is a "black-ball" stool (Opium), and that is getting better. Our Sulph. bottle has been reloaded at least fifty times in the past six years, since I put the graft Dr. Swan sent me in with the initial charge of No. 5 pellets. I cured a case of ringworm, that resisted a Texas doctor's salve, by one dose of Tuberculinum.

The ringworm was on back of hands and dorsum of feet. Itching worse after midnight, worse by scratching—voluptuous itching. After midnight he woke up with a cold, clammy sweat all over. Tuberculinum$^{30}$, one dose. Prescription made was non-homœopathic; purely experimental.

* Postscript to a personal letter to the editor.

